# Mobile phone interventions for tuberculosis should ensure access to mobile phones to enhance equity – a prospective, observational cohort study in Peruvian shantytowns

**DOI:** 10.1111/tmi.13087

**Published:** 2018-06-22

**Authors:** Matthew J. Saunders, Tom Wingfield, Marco A. Tovar, Niamh Herlihy, Claudio Rocha, Karine Zevallos, Rosario Montoya, Eric Ramos, Sumona Datta, Carlton A. Evans

**Affiliations:** ^1^ Infectious Diseases & Immunity Imperial College London Wellcome Trust Imperial College Centre for Global Health Research London UK; ^2^ Innovación Por la Salud Y Desarrollo (IPSYD) Asociación Benéfica PRISMA Lima Perú; ^3^ Innovation for Health and Development (IFHAD) Laboratory of Research and Development Universidad Peruana Cayetano Heredia Lima Perú; ^4^ Clinical Infection, Microbiology, and Immunity Institute of Infection and Global Health University of Liverpool Liverpool UK; ^5^ Social Medicine, Infectious diseases and Migration Group Department of Public Health Science Karolinksa Institutet Stockholm Sweden; ^6^ LIV‐TB Liverpool School of Tropical Medicine Liverpool UK

**Keywords:** tuberculosis, Mhealth, Mobile Health, ehealth, tuberculose, m‐santé, santé mobile, e‐santé

## Abstract

**Objectives:**

Mobile phone interventions have been advocated for tuberculosis care, but little is known about access of target populations to mobile phones. We studied mobile phone access among patients with tuberculosis, focusing on vulnerable patients and patients who later had adverse treatment outcomes.

**Methods:**

In a prospective cohort study in Callao, Peru, we recruited and interviewed 2584 patients with tuberculosis between 2007 and 2013 and followed them until 2016 for adverse treatment outcomes using national treatment registers. Subsequently, we recruited a further 622 patients between 2016 and 2017. Data were analysed using logistic regression and by calculating relative risks (RR).

**Results:**

Between 2007 and 2013, the proportion of the general population of Peru without mobile phone access averaged 7.8% but for patients with tuberculosis was 18% (*P* < 0.001). Patients without access were more likely to hold a lower socioeconomic position, suffer from food insecurity and be older than 50 years (all *P* < 0.01). Compared to patients with mobile phone access, patients without access at recruitment were more likely to subsequently have incomplete treatment (20% *vs*. 13%, RR = 1.5; *P* = 0.001) or an adverse treatment outcome (29% *vs*. 23% RR = 1.3; *P* = 0.006). Between 2016 and 2017, the proportion of patients without access dropped to 8.9% overall, but remained the same (18%) as in 2012 among the poorest third.

**Conclusion:**

Access to mobile phones among patients with tuberculosis is insufficient, and rarest in patients who are poorer and later have adverse treatment outcomes. Thus, mobile phone interventions to improve tuberculosis care may be least accessed by the priority populations for whom they are intended. Such interventions should ensure access to mobile phones to enhance equity.

## Abstract (fr)

**Objectifs:**

Les interventions basées sur la téléphonie mobile ont été préconisées pour les soins de la tuberculose (TB), mais on en sait peu sur l'accès des populations cibles aux téléphones mobiles. Nous avons étudié l'accès aux téléphones mobiles parmi les patients souffrant de tuberculose, en mettant l'accent sur les patients vulnérables et les patients qui ont plus tard eu des résultats adverses.

**Méthodes:**

Dans une étude de cohorte prospective à Callao, au Pérou, nous avons recruté et interviewé 2.584 patients atteints de TB entre 2007 et 2013 et les avons suivis jusqu'en 2016 pour des résultats thérapeutiques adverses en utilisant les registres de traitement nationaux. Par la suite, nous avons recruté 622 patients supplémentaires entre 2016 et 2017. Les données ont été analysées en utilisant la régression logistique et en calculant les risques relatifs (RR).

**Résultats:**

Entre 2007 et 2013, la proportion de la population générale du Pérou sans accès au téléphone mobile était en moyenne de 7,8%, mais pour les patients avec la TB, elle était de 18% (*p* <0,001). Les patients sans accès étaient plus susceptibles de faire partie de la position socioéconomique inférieure, de souffrir d'insécurité pour la nourriture et d'avoir plus de 50 ans (tous *p* <0,01). Comparés aux patients ayant un accès au téléphone mobile, les patients sans accès lors du recrutement étaient plus susceptibles d'avoir par la suite un traitement incomplet (20% contre 13%, RR = 1,5; *p* = 0,001) ou un résultat de traitement adverse (29% contre 23% RR = 1,3; *p* = 0,006). Entre 2016 et 2017, la proportion de patients sans accès a chuté à 8,9% globalement, mais restait la même (18%) qu'en 2012 parmi le tiers le plus pauvre.

**Conclusion:**

L'accès à la téléphonie mobile chez les patients atteints de TB est insuffisant et plus rare chez les patients plus pauvres qui ont ensuite des résultats de traitement adverses. Ainsi, les interventions basées sur la téléphonie mobile visant à améliorer les soins antiTB peuvent être moins accessibles par les populations prioritaires auxquelles elles sont destinées. De telles interventions devraient garantir l'accès aux téléphones mobiles pour renforcer l’équité.

## Introduction

In 2016, tuberculosis (TB) was estimated to have killed 1.6 million people and ranked as the leading cause of death from an infectious disease worldwide 1. TB continues to disproportionately affect the poorest members of society, and socioeconomic factors are associated with higher risk of TB disease, diagnostic delay and adverse TB treatment outcomes 2, 3. The great majority of deaths associated with TB are believed to be preventable if people can promptly and effectively access adequate health services.

In its End TB Strategy, WHO highlights the importance of patient‐centred care to improve diagnosis and reduce adverse treatment outcomes 4. To make the ambitious vision set out in this strategy a reality, innovative and low‐cost solutions are required to complement traditional National TB Program (NTP) approaches and ensure that the most socioeconomically disadvantaged populations use available medical care 5.

Due to the pace with which information technology has developed, Digital Health, including mobile phone interventions, has become an increasingly popular idea among policy makers, health system managers and public health researchers. In 2015, WHO established a Global Task Force on Digital Health for TB and, together with the European Respiratory Society, published an agenda detailing how digital health interventions could be promoted and integrated into national operational plans to implement the End TB Strategy 6, 7. During this consultation, several ‘target product profiles’ were developed to define the features of desired digital health solutions and stimulate further interest from potential developers 8. These include interventions to improve patient care; support disease surveillance and monitoring; facilitate program management; and provide platforms for eLearning.

Despite the wealth of interest surrounding information technology, there is a shortage of published evidence assessing the feasibility and effectiveness of mobile phone interventions for patient care. In this study, we aimed to assess rates of mobile phone access among patients with TB, focusing on vulnerable patients and patients who later had adverse treatment outcomes.

## Methods

### Study design, setting and participants

We conducted an open, prospective cohort study of patients with TB in 15 desert shantytown communities in Ventanilla, Callao, Peru. Participants were recruited from December 2007 until December 2013 and followed‐up until January 2016. Until 2011, participants were concurrently recruited to the Innovative Socioeconomic Interventions Against Tuberculosis (ISIAT) project that evaluated socioeconomic support provided to TB‐affected households 9. Ventanilla is an area of considerable poverty with a large population of migrants and internally displaced people from the mountainous, costal and jungle areas of Peru 10, 11. In this setting, TB was diagnosed and treated almost exclusively in government‐run health posts, free of direct charges. Treatment constituted community clinic‐based directly observed therapy (DOT) for every dose. The actual TB case notification rate in these health posts collected collaboratively during the study period was 183/100 000 people. Inclusion criteria were patients with a new diagnosis of TB who were registered to receive treatment in health posts. Exclusion criteria were being unwilling or unable to provide informed written consent (and for minors able to do so, also their assent), or not completing the initial interview. Study approvals included the internationally accredited ethics committee of the Asociación Benéfica PRISMA, Perú and the Peruvian NTP.

### Initial procedures

Study personnel and participants interacted at household visits and health posts. Participants completed a locally developed questionnaire in Spanish characterising demographics, household assets, access to essential services, food insecurity and work status. In rare cases when the participant only spoke Quechua, the questionnaire was completed with a Spanish‐speaking relative acting as a translator. Food insecurity was defined as going to bed hungry because of shortage of food on at least one night in the month prior to recruitment. This study assessed access to mobile phones, but did not in any way influence access to them. Furthermore, we believe that there are no other such interventions in this setting. Access to a mobile phone was defined if the patient (or for minors their guardian) reported access, in their household, to at least one working mobile phone able to send and receive text messages. National statistics on mobile phone subscriptions were accessed from International Telecommunication Union statistics 12. At recruitment, all participants were requested to give a sputum sample, which was tested using Ziehl‐Nielsen microscopy and the microscopic‐observation drug‐susceptibility (MODS) assay, a test not always routinely available in Peru during the study period. Participants were defined as having multidrug‐resistant (MDR) TB if they were prescribed an MDR‐TB treatment regimen, or if sputum testing confirmed TB resistance to isoniazid and rifampicin.

### Follow‐up

TB treatment outcomes and TB recurrence were ascertained using health post‐treatment registers collected until January 2016. We visited participant's households on average three years after recruitment to corroborate these outcomes and ask about TB recurrence treated outside the study area. Adverse treatment outcomes were defined according to national guidelines as: treatment failure; incomplete treatment (no therapy taken for at least 30 consecutive days, which is locally termed ‘abandoned treatment’); death from any cause during treatment; and TB recurrence (defined as a new diagnosis of TB after treatment success within 24 months of starting treatment for the original TB episode). Good treatment outcome referred to a participant being cured or having completed treatment without known recurrence within 24 months of starting treatment. Participants who were transferred to another health post‐outside of the study site, or were lost to follow‐up but not considered to have abandoned treatment, were counted as having an unknown outcome.

### Analysis

We used Stata (StataCorp, College Station, TX) and *P* values were two‐sided with significance assessed at the 5% level. All recruited patients with TB were included in the analysis. The distributions of continuous data were assessed and non‐Gaussian data were summarised by medians and interquartile ranges (IQR). Categorical or binary data were summarised as proportions. We defined three age categories: 15–50 years as the working age population in this setting; <15 years as children and adolescents; and >50 years as older adults, who are at increased risk of TB in this setting 13. Because this project was based in an almost universally impoverished region, we used principal component analysis to develop a household ‘poverty’ socioeconomic position (SEP) index incorporating 18 variables, including participant education level, into a single continuous variable and defined three SEP terciles of equal population size (poor SEP, poorer SEP and poorest SEP) 14. Associations between mobile phone access, SEP and year of recruitment were investigated using the chi‐squared test for trend and were plotted against national estimates of mobile phone access during the study period. Our estimates of mobile phone access in 2008 included the few patients who were recruited in December 2007. Factors associated with being without mobile phone access at recruitment were investigated using univariable logistic regression to calculate odds ratios (OR) with 95% confidence intervals (CI) for the variables in Table 1. We then built a multivariable logistic regression model including all variables plausibly associated with mobile phone access, including year of recruitment, to estimate adjusted OR (aOR). Subsequently, we investigated whether being without mobile phone access at the time of recruitment was associated with adverse treatment outcomes by calculating unadjusted relative risks (RR).

**Table 1:** Baseline characteristics of patients with tuberculosis recruited between 2007–2013 in Callao, Peru and analysis of factors associated with being without access to a mobile phone

Variable	All (*n* = 2584)	With mobile phone access (*n* = 2106)	Without mobile phone access (*n* = 478)	OR for being without access to a mobile phone			
				Univariable analysis		Multivariable analysis^e^ (*n* = 2526)	
				OR (95% CI)	*P* value	aOR (95% CI)	*P* value
Male sex (*n*, %)	1587 (61)	1288 (61)	299 (63)	1.1 (0.86–1.3)	0.6		
Age (median; IQR)	28 (21–42)	28 (21–41)	32 (21–50)	NA	NA		
Age group							
Working age: 15–50 years (*n*, %)	2011 (78)	1678 (80)	333 (70)	Reference	Reference	Reference	Reference
Children and adolescents: <15 years (*n*, %)	134 (5)	105 (5)	29 (6)	1.4 (0.91–2.1)	0.1	1.2 (0.77–1.9)	0.4
Older age: >50 years (*n*, %)	439 (17)	323 (15)	116 (25)	1.8 (1.4–2.3)	<0.001	1.9 (1.5–2.5)	<0.001
Work status^a^							
Formal paid work (*n*, %)	370 (15)	319 (15)	51 (11)	Reference	Reference		
Family or informal paid work (*n*, %)	595 (23)	474 (22)	121 (25)	1.6 (1.1–2.3)	0.01		
Domestic work (*n*, %)	472 (19)	381 (18)	91 (20)	1.5 (1.0–2.2)	0.04		
Too sick to work (*n*, %)	493 (20)	393 (19)	100 (21)	1.6 (1.1–2.3)	0.01		
Student, child or other (*n*, %)	613 (24)	504 (24)	109 (23)	1.4 (0.95–1.9)	0.10		
Socioeconomic position^d^ tercile							
Poor (*n*, %)	847 (33)	756 (36)	91 (19)	Reference	Reference	Reference	Reference
Poorer (*n*, %)	860 (33)	722 (34)	138 (30)	1.6 (1.2–2.1)	0.001	1.6 (1.2–2.1)	0.002
Poorest (*n*, %)	877 (34)	628 (30)	249 (52)	3.3 (2.5–4.3)	<0.001	3.0 (2.3–4.0)	<0.001
Food insecurity^b^ (*n*, %)	553 (22)	412 (20)	141 (30)	1.7 (1.4–2.1)	<0.001	1.5 (1.2–1.9)	0.001
No health insurance^c^ (*n*, %)	428 (21)	336 (20)	92 (25)	1.3 (1.0–1.8)	0.03		
Multi‐drug resistant tuberculosis (*n*, %)	197 (7.6)	160 (7.6)	30 (7.7)	1.0 (0.70–1.5)	0.9		
Sputum‐smear positive tuberculosis (*n*, %)	1419 (55)	1154 (55)	265 (55)	1.0 (0.84–1.3)	0.80		

OR, odds ratio; aOR, adjusted odds ratio; IQR, inter‐quartile range.

a Data were available for 2543 (95%) participants.

b Data were available for 2526 (98%) participants.

c Data were available for 2017 (78%) participants.

d The variables included in our socioeconomic position index were: home ownership, wall material, floor material, water supply, type of toilet, cooking fuel, lighting supply, education levels of the male and female heads of household; and owning a TV, food processor, wardrobe, radio, fridge, stove, iron, landline telephone and coffee maker.

e The final model was also adjusted for year of recruitment.

### Validation: current rates of access

As our analysis demonstrated mobile phone access significantly increased during the study period, we evaluated current rates of mobile phone access by performing an interim analysis of data collected during the study PREVENT TB: improving determinants of TB cure, prevention and diagnosis 15. Patients with TB were recruited from 32 communities in Callao using the same eligibility criteria and measurements as the original cohort, with data analysed as described above to investigate the association between mobile phone access and SEP for this cohort. These communities included the 15 that were studied in the original cohort.

## Results

### Initial data

**Figure 1:**
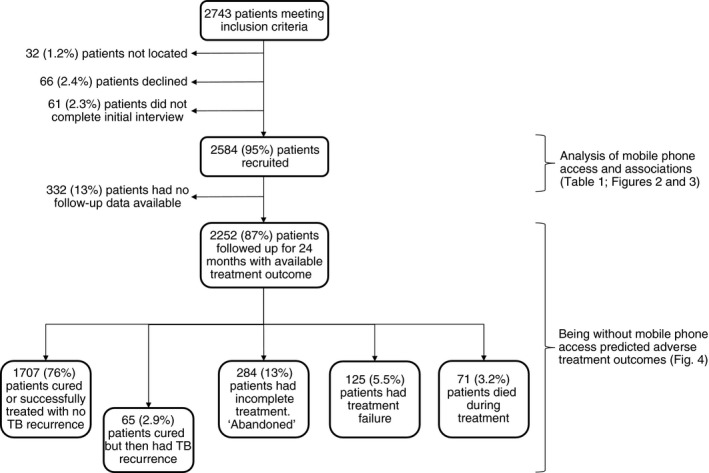
A prospective cohort study of patients with tuberculosis in Callao, Peru, 2007–2013 with follow‐up until 2016.

As presented in Table 1 and Figure 1, health post‐treatment records showed that 2743 patients met the inclusion criteria. We located 2711 (99%) and recruited 2584 (95%) of them. Sixty‐six (2.4%) declined and 61 (2.3%) were ineligible because they did not complete the initial interview. A total of 1587 (61%) patients were male, and the median age was 28 (IQR = 21–43). One hundred and ninety‐seven (7.6%) patients were defined as having MDR‐TB.

### Mobile phone access

**Figure 2:**
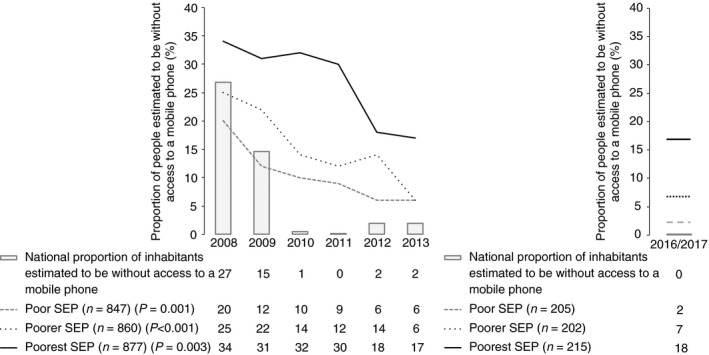
The association between patients with tuberculosis without mobile phone access, socioeconomic position (SEP) and time plotted against estimates of national mobile phone access. *P* values represent a chi‐squared test for trend between mobile phone access and year of recruitment. (a) Original cohort (*n* = 2584) 2007–2013. (b) Validation (*n* = 622): 2016–2017.

Figure 2 shows that at recruitment 478 (18%) patients were without access to a working mobile phone. This rate decreased during the study for all patients (*P* < 0.001); and for patients who were poorest (*P* = 0.003), poorer (*P* = <0.001) and poor (*P* = 0.001). The proportion of patients with TB who were without mobile phone access was higher than the national estimate of the proportion of Peru's general population without mobile phone access (which averaged 7.8% during study recruitment; *P* < 0.001). This difference was most marked among the poorest tercile of patients with TB.

### Associations with mobile phone access

**Figure 3:**
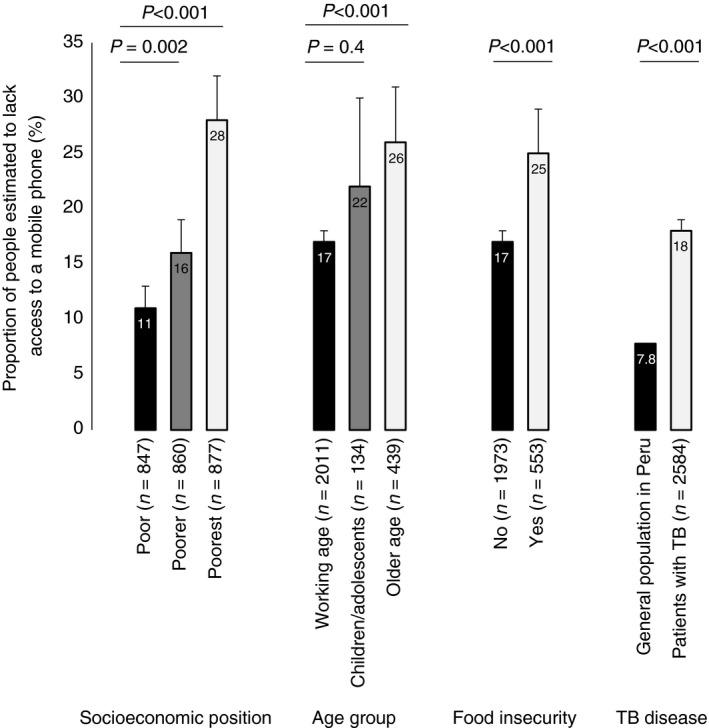
The association between patients with tuberculosis (TB) (*n* = 2584) without mobile phone access, socioeconomic position (SEP), age, food insecurity, and compared with the general population. *P* values are adjusted multivariable analysis (Table 1) between these factors and mobile phone access and for the comparison of patients with TB *vs*. the general population, a two‐sample proportion test. Error bars represent 95% confidence intervals.

As can be seen in Table 1 and Figure 3, in univariable analysis, patients without mobile phone access had lower SEP (poorer *vs*. poor SEP OR = 1.6, *P* = 0.001; poorest *vs*. poor SEP OR = 3.3, *P* < 0.001) and were more likely to suffer food insecurity (OR = 1.7, *P* < 0.001); not have health insurance (OR = 1.3, *P* = 0.03); be an older adult (OR = 1.8, *P* < 0.001) and not be in formal work (Table 1). Multivariable analysis demonstrated that being without mobile phone access was independently associated with SEP (poorer *vs*. poor SEP aOR = 1.6, *P* = 0.002; poorest *vs*. poor SEP aOR = 3.0, *P* < 0.001), being an older adult (aOR = 1.9, *P* < 0.001) and food insecurity (aOR = 1.5, *P* = 0.001). Figure 3 shows mobile phone access for each of the statistically significant independent associations.

### Follow‐up

**Figure 4:**
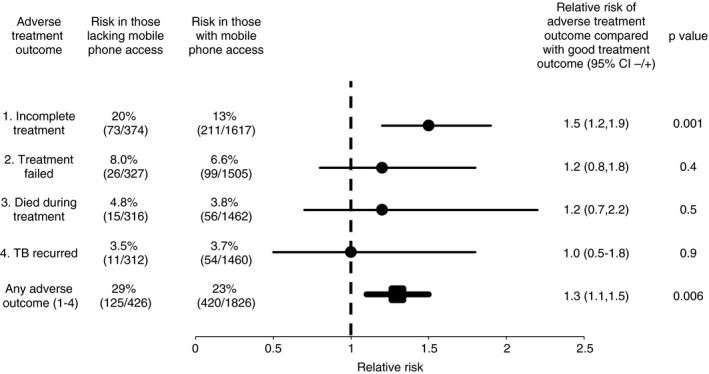
Being without mobile phone access at the time of tuberculosis (TB) diagnosis predicted adverse treatment outcomes. About 87% of patients with TB (*n* = 2252) had a defined treatment outcome available for analysis.

Treatment outcome was defined for 2252/2584 (87%) patients; for similar proportions of patients without *vs*. with access to a mobile phone (426/458, 89% *vs*. 1826/2106, 87%, respectively, *P* = 0.2) (Figure 4). Overall, 545/2252 (24%) patients had an adverse treatment outcome. Of these, 284 (52%) had incomplete treatment; 125 (23%) had treatment failure; 71 (13%) died during treatment; and 65 (12%) were initially classified as cured or having completed treatment but then had TB recurrence. Patients without mobile phone access had a higher risk of incomplete treatment *vs*. good outcome (20% *vs*. 13%; RR = 1.5, *P* = 0.001). Mobile phone access was not significantly associated with risk of treatment failure (8.0% *vs*. 6.6%; RR = 1.2, *P* = 0.4), death (4.8% *vs*. 3.8%; RR = 1.2, *P* = 0.5) or TB recurrence (3.5% *vs*. 3.7%; RR = 1.0, *P* = 0.9) *vs*. good outcome. Overall, patients without mobile phone access were significantly more likely to experience an adverse treatment outcome (29% *vs*. 23%, respectively; RR = 1.3, *P* = 0.006).

### Validation: current rates of access

Between July 2016 and March 2017, we recruited 622/683 (91%) patients with TB to the ongoing PREVENT TB study. Overall, 55/622 (8.9%) patients were without access to a mobile phone at recruitment. We observed a similar distribution of mobile phone access by SEP, with 38/215 (18%) patients from the poorest SEP being without access to a mobile phone *vs*. 14/202 (6.9%) and 3/205 (1.5%) from poorer and poor SEP, respectively (*P* < 0.001).

## Discussion

Mobile phone interventions aim to widen access to health care with the ultimate objectives in TB control of improving diagnosis, adherence to treatment (including preventive treatment) and outcomes. In this prospective cohort study in Peruvian desert shantytowns, the proportion of patients with TB without access to a mobile phone was high and varied substantially with several important socioeconomic factors. Patients without access to a mobile phone were poorer, older, more likely to suffer food insecurity, and were significantly more likely to experience an adverse treatment outcome than their counterparts who had access to mobile phones. Despite an increase in access to mobile phones in recent years, our current data from the PREVENT TB study demonstrate that access remains far from universal, with approximately one in five of the poorest patients not having access to a mobile phone at the time they were diagnosed. Our results therefore suggest that mobile phone interventions relying on patient mobile phone access as a prerequisite to eligibility are likely to relatively neglect the poorest patients at highest risk of adverse treatment outcomes. To our knowledge, this is the first study to describe these issues; we have not been able to identify any other studies making comparable analyses.

Whilst there is significant interest in the potential of mobile phone‐based interventions to improve TB care, rigorous evidence on their impact is limited. Much of the enthusiasm for mobile phone interventions assumes high anticipated rates of access to mobile phones among patients, positive experiences extrapolated from outside TB care, and a body of evidence suggesting mobile phone interventions would be acceptable to patients 16, 17, 18, 19. Furthermore, reminder systems, such as telephone calls and letters, have been shown to improve patient attendance at TB clinic appointments for diagnosis and treatment 20. However, the findings of recent reviews on the potential for mobile phone interventions, including daily text message reminders, to influence patient treatment outcomes were inconclusive and the authors repeatedly highlighted the lack of high‐quality data available for analysis 21, 22. Outside of TB care, a recent study in Kenya evaluating text message reminders for increasing vaccination coverage showed no increase in overall vaccination rates in the group only allocated to receive reminders 23. These ‘nudge’ interventions have historically been popular with policy makers partly because they are perceived to be wide‐reaching and low‐cost. In fact, the evidence suggests that mobile phone interventions are no so‐called silver bullet and more research is required demonstrating their effectiveness, equity and safety before widespread implementation may be recommended. Several randomised controlled trials assessing the impact of more complex mobile phone interventions on TB cure rates and preventive treatment adherence rates are ongoing and will contribute to this evidence 24, 25, 26. Such interventions may have more success in improving preventive treatment adherence rates as, in contrast to TB treatment, this is typically self‐administered and is an area in need of significant improvement 27.

Poverty and inequality have long been recognised as important drivers of the TB epidemic with relatively poorer patients suffering a disproportionate burden of disease, adverse treatment outcomes and TB‐related catastrophic costs 28, 29, 30. Mitigating socioeconomic factors and reducing inequality in health care have recently been formally consolidated in the WHO End TB Strategy, which for the first time in TB control gives precedence to interventions aiming to reduce poverty, ensure food security and improve equity of access to health services among vulnerable groups. Accordingly, our findings, which suggest that mobile phone interventions relying on patient mobile phone access may relatively neglect such vulnerable groups and therefore potentially widen the inequality gap, have important implications for public health practitioners and TB policy makers as they design and scale‐up interventions. Specifically, mobile phone interventions that aim to deliver social protection schemes to patients with TB, such as cash transfers, should ensure patients have access to mobile phones to avoid inadvertently neglecting the very patients that they are most designed to help 31, 32.

In addition to the findings reported in this study, our experience of working in resource‐constrained, desert shantytowns in Peru has alerted us to several caveats to using mobile phones as a means of communicating with patients with TB. In our setting, mobile phones are not a secure, confidential and safe means of communication. Firstly, patients with TB have highlighted the experience of stigma and discrimination when others have read their TB‐related text messages, although this may be avoided using alternative strategies such as password‐protected messages 33, 34. Secondly, handsets are frequently prone to damage, faults and especially theft. Thirdly, some patients may share their phone, have more than one phone, change their number and their network provider during treatment or have problems maintaining battery charge due to intermittent access to electrical supply. Even if overall rates of mobile phone access are high, these logistical problems challenge the practicality and reliability of communicating with patients through mobile phones sustainably throughout TB treatment, as this takes at least six months.

Our study has several limitations. Firstly, we were unable to account for changes in mobile phone access over the course of each patient's illness. A high proportion of patients with TB in Peru incur TB‐related catastrophic costs and report dissaving behaviours to cope with the financial burden associated with their disease 29, 35. This may mean that when challenged by TB‐associated direct and indirect costs they must sell their phone, give up their network subscription or not have enough phone credit to communicate during the time that they are unwell. Furthermore, we did not collect data on type of handset (including tablet computers); handset sharing; network and/or internet access; or the proportion of people who experienced theft, damage or faults. These data would have been useful because many mobile phone interventions, including video‐observed therapy, rely on relatively expensive smart phones with Internet and social media access that we believe to be rarely available in our setting and particularly prone to theft. The strengths of this study include our high recruitment rate minimising the risk of selection bias, our detailed baseline interviews, our large sample size and our collaboration with government‐run health posts to ascertain treatment outcomes and TB recurrence. However, we may have underestimated the number of patients suffering TB recurrence as some may have received treatment outside of the jurisdiction of the study, although this risk was reduced by our active follow‐up of participants.

During the study period, Peru experienced substantial economic growth and the number of people living in absolute poverty dropped significantly 36. This is reflected by the rising rates of mobile phone access throughout the study, suggesting that mobile phone access increases with economic growth. However, our recent data from 2016 to 2017 demonstrate that these benefits have not been felt equally across the whole population, with the proportion of people without access to a mobile phone among the poorest third of patients remaining the same in 2016–2017 as it was in 2012. Our results therefore have particular relevance for other resource constrained, urban settings that are not experiencing such rapid development and where mobile phone access, especially among impoverished and marginalised patients with TB, is likely to be lower and less equitable. This is also true for rural areas that are less likely to have mobile phone service coverage. Finally, it should be emphasised that our findings represent association and not causation between mobile phone access and adverse treatment outcome. We did not intend to demonstrate an independent, causal effect of mobile phones on adverse treatment outcomes and believe that the observed association is mediated by poverty and the associated catastrophic costs, marginalisation and disempowerment that hamper TB control 37.

In conclusion, our results reveal that being without access to a mobile phone is more frequent among patients with TB than the general population and is particularly frequent in people with several important indicators of vulnerability, including later adverse treatment outcomes. Thus, mobile phone interventions may be least accessed by the high‐risk groups that are priorities for strengthening TB care and prevention. Further evidence is required to evaluate the feasibility, equity and effectiveness of mobile phone interventions for TB control. Whilst such studies are performed, existing mobile phone interventions should ensure access to mobile phones among participants to enhance equity.

## Funding

This research and members of the research team were funded by: The Wellcome Trust (awards 057434/Z/99/Z, 070005/Z/02/Z, 078340/Z/05/Z, 105788/Z/14/Z, 209075/Z/17/Z and 201251/Z/16/Z); DFID‐CSCF; the Joint Global Health Trials Consortium (MRC, DFID, & Wellcome Trust award MR/K007467/1); the Bill and Melinda Gates Foundation (award OPP1118545); Imperial College National Institutes of Health Research Biomedical Research Centre; the Foundation for Innovative New Diagnostics (FIND); the Sir Halley Stewart Trust; the World Health Organization; the STOP TB partnership's TB REACH initiative funded by the Government of Canada and the Bill & Melinda Gates Foundation (W5_PER_CDT1_PRISMA); The Academy of Medical Sciences; and the charity IFHAD: Innovation For Health And Development. None of these organisations had any role in or placed any restrictions on the preparation or publication of this manuscript. This manuscript represents the research and opinion of the authors and not of any of these funding organisations.

## References

[1] World Health Organization . Global Tuberculosis Report. 2017.

[2] Saunders MJ , Evans CA . Fighting poverty to prevent tuberculosis. Lancet Infect Dis 2016: 16: 395–396. 26725447 10.1016/S1473-3099(15)00434-X

[3] Bonadonna LV , Saunders MJ , Zegarra R *et al* Why wait? The social determinants underlying tuberculosis diagnostic delay. PLoS ONE 2017: 12: e0185018. 28945782 10.1371/journal.pone.0185018 PMC5612650

[4] Uplekar M , Weil D , Lonnroth K *et al* WHO's new end TB strategy. Lancet 2015: 385: 1799–1801. 25814376 10.1016/S0140-6736(15)60570-0

[5] Lönnroth K , Castro KG , Chakaya JM *et al* Tuberculosis control and elimination 2010‐50: cure, care, and social development. Lancet 2010: 375: 1814–1829. 20488524 10.1016/S0140-6736(10)60483-7

[6] World Health Organization . Digital health for the End TB strategy: an agenda for action. 2015.

[7] World Health Organization . Global Task Force on digital health for TB. (Available from: http://www.who.int/tb/areas-of-work/digital-health/global-task-force/en/) [18 Mar 2017]

[8] Falzon D , Timimi H , Kurosinski P *et al* Digital health for the End TB strategy: developing priority products and making them work. Eur Respir J 2016: 48: 29–45. 27230443 10.1183/13993003.00424-2016 PMC4929075

[9] Rocha C , Montoya R , Zevallos K *et al* The Innovative Socio‐economic Interventions Against Tuberculosis (ISIAT) project: an operational assessment. Int J Tuberc Lung Dis 2011: 15: S50–S57. 10.5588/ijtld.10.0447 PMC3160483 21740659

[10] Instituto Nacional de Estadistica e Informatica . Peru: Migración interna reciente y el sistema de ciudades 2002–2007. Lima: 2011.

[11] Instituto Nacional de Estadistica e Informatica . Mapa de Pobreza Provincial y Distrital 2013. Lima: 2015.

[12] International Telecommunication Union . End‐2016 estimates for key ICT indicators. 2016. (Available from: http://www.itu.int/en/ITU-D/Statistics/Documents/statistics/2016/ITU_Key_2005-2016_ICT_data.xls) [1 Jan 2016]

[13] Saunders MJ , Wingfield T , Tovar MA *et al* A score to predict and stratify risk of tuberculosis in adult contacts of tuberculosis index cases: a prospective derivation and external validation cohort study. Lancet Infect Dis 2017: 17: 1190–1199. 28827142 10.1016/S1473-3099(17)30447-4 PMC7611139

[14] Boccia D , Hargreaves J , de Stavola BL *et al* The association between household socioeconomic position and prevalent tuberculosis in Zambia: a case‐control study. PLoS ONE 2011: 6: e20824. 21698146 10.1371/journal.pone.0020824 PMC3117783

[15] PREVENT TB: Improving determinants of TB cure, prevention & diagnosis. (Available from: 10.1186/isrctn17820976) [6 Feb 2018]

[16] Horvath T , Azman H , Kennedy GE , Rutherford GW . Mobile phone text messaging for promoting adherence to antiretroviral therapy in patients with HIV infection. Cochrane Database Syst Rev 2012: Issue 3: Art. No.: CD009756. 10.1002/14651858.cd009756. PMC6486190 22419345

[17] Whittaker R , McRobbie H , Bullen C , Rodgers A , Gu Y . Mobile phone‐based interventions for smoking cessation. Cochrane Database Syst Rev 2016: Issue 4: Art. No.: CD006611. 10.1002/14651858.cd006611.pub4. PMC6485940 27060875

[18] Iribarren S , Beck S , Pearce PF *et al* TextTB: a mixed method pilot study evaluating acceptance, feasibility, and exploring initial efficacy of a text messaging intervention to support TB treatment adherence. Tuberc Res Treat 2013: 2013: 1–12. 10.1155/2013/349394 PMC3876704 24455238

[19] Free C , Phillips G , Galli L *et al* The effectiveness of mobile‐health technology‐based health behaviour change or disease management interventions for health care consumers: a systematic review. PLoS Med 2013: 10: e1001362. 23349621 10.1371/journal.pmed.1001362 PMC3548655

[20] Liu Q , Abba K , Alejandria MM , Sinclair D , Balanag VM , Lansang MAD . Reminder systems to improve patient adherence to tuberculosis clinic appointments for diagnosis and treatment. Cochrane Database Syst Rev 2014: Issue 11: Art. No.: CD006594. 10.1002/14651858.cd006594.pub3 PMC4448217 25403701

[21] Nglazi MD , Bekker L‐G , Wood R *et al* Mobile phone text messaging for promoting adherence to anti‐tuberculosis treatment: a systematic review. BMC Infect Dis 2013: 13: 566. 24295439 10.1186/1471-2334-13-566 PMC4219402

[22] Ngwatu BK , Nsengiyumva NP , Oxlade O *et al* The impact of digital health technologies on tuberculosis treatment: a systematic review. Eur Respir J 2018: 51: 1701596. 29326332 10.1183/13993003.01596-2017 PMC5764088

[23] Gibson DG , Ochieng B , Kagucia EW *et al* Mobile phone‐delivered reminders and incentives to improve childhood immunisation coverage and timeliness in Kenya (M‐SIMU): a cluster randomised controlled trial. Lancet Glob Health 2017: 5: e428–e438. 28288747 10.1016/S2214-109X(17)30072-4 PMC5348605

[24] Bediang G , Stoll B , Elia N *et al* SMS reminders to improve the tuberculosis cure rate in developing countries (TB‐SMS Cameroon): a protocol of a randomised control study. Trials 2014: 15: 35. 24460827 10.1186/1745-6215-15-35 PMC3902069

[25] van der Kop ML , Memetovic J , Patel A *et al* The effect of weekly text‐message communication on treatment completion among patients with latent tuberculosis infection: study protocol for a randomised controlled trial (WelTel LTBI). BMJ Open 2014: 4: e004362. 10.1136/bmjopen-2013-004362 PMC3987735 24719431

[26] Bassett IV , Giddy J , Chaisson CE *et al* A randomized trial to optimize HIV/TB care in South Africa: design of the Sizanani trial. BMC Infect Dis 2013: 13: 390. 23972276 10.1186/1471-2334-13-390 PMC3765953

[27] Saunders MJ , Tovar MA , Datta S *et al* Pragmatic tuberculosis prevention policies for primary care in low‐ and middle‐income countries. Eur Respir J 2018: 51: 1800315. 29567728 10.1183/13993003.00315-2018

[28] Lönnroth K , Jaramillo E , Williams BG *et al* Drivers of tuberculosis epidemics: the role of risk factors and social determinants. Soc Sci Med 2009: 68: 2240–2246. 19394122 10.1016/j.socscimed.2009.03.041

[29] Wingfield T , Boccia D , Tovar M *et al* Defining catastrophic costs and comparing their importance for adverse tuberculosis outcome with multi‐drug resistance: a prospective cohort study, Peru. PLoS Med 2014: 11: e1001675. 25025331 10.1371/journal.pmed.1001675 PMC4098993

[30] Westerlund EE , Tovar MA , Lönnermark E *et al* Tuberculosis‐related knowledge is associated with patient outcomes in shantytown residents; Results from a cohort study, Peru. J Infect 2015: 71: 347–357. 26033695 10.1016/j.jinf.2015.05.010 PMC7617110

[31] Wingfield T , Boccia D , Tovar MA *et al* Designing and implementing a socioeconomic intervention to enhance TB control: operational evidence from the CRESIPT project in Peru. BMC Public Health 2015: 15: 810. 26293238 10.1186/s12889-015-2128-0 PMC4546087

[32] Wingfield T , Tovar MA , Huff D *et al* A randomized controlled study of socioeconomic support to enhance tuberculosis prevention and treatment, Peru. Bull World Health Organ 2017: 95: 270–280. 28479622 10.2471/BLT.16.170167 PMC5407248

[33] Acosta C , Baldwin M , Montoya R *et al* TB stigmatization is associated with treatment non‐adherence in impoverished areas of Peru. Int J Tuberc Lung Dis 2010: 13: S193.

[34] Albino S , Tabb KM , Requena D *et al* Perceptions and acceptability of short message services technology to improve treatment adherence amongst tuberculosis patients in Peru: a focus group study. PLoS ONE 2014: 9: e95770. 24828031 10.1371/journal.pone.0095770 PMC4020740

[35] Wingfield T , Tovar MA , Huff D *et al* The economic effects of supporting tuberculosis‐affected households in Peru. Eur Respir J 2016: 48: 1396–1410. 27660507 10.1183/13993003.00066-2016 PMC5091496

[36] The World Bank . World Bank: Country, Peru. (Available from: http://data.worldbank.org/country/peru) [6 Feb 2018]

[37] Wingfield T , Tovar MA , Datta S *et al* Addressing social determinants to end tuberculosis. Lancet 2018: 391: 1129–1132. 29595481 10.1016/S0140-6736(18)30484-7 PMC7611140

